# The value of MRI compared to conventional radiography in analysing morphologic changes in the spine in axial spondyloarthritis

**DOI:** 10.1186/s13244-021-01127-x

**Published:** 2021-12-11

**Authors:** Gabriel Adelsmayr, Andreas Haidmayer, Christopher Spreizer, Michael Janisch, Franz Quehenberger, Eva Klocker, Winfried Graninger, Michael Fuchsjäger, Josef Hermann

**Affiliations:** 1 Division of General Radiology, Department of Radiology, Medical University of Graz, Auenbruggerplatz 9, 8036 Graz, Austria; 2 Division of Rheumatology and Immunology, Department of Internal Medicine, Medical University of Graz, Auenbruggerplatz 15, 8036 Graz, Austria; 3 Department of Internal Medicine, Hospital Southern Styria, Dr.-Schwaiger-Straße 1, 8490 Bad Radkersburg, Austria; 4 Southeastern CT-Institute, Gleichenberger Str. 35, 8330 Feldbach, Austria; 5 Institute for Medical Informatics, Statistics and Documentation, Medical University of Graz, Auenbruggerplatz 2/9/V, 8036 Graz, Austria; 6 Division of Oncology, Department of Internal Medicine, Medical University of Graz, Auenbruggerplatz 15, 8036 Graz, Austria

**Keywords:** Radiography, Magnetic resonance imaging, Spondyloarthritis, Observer variation

## Abstract

**Background:**

Imaging of morphologic changes in the vertebral spine in axial spondyloarthritis (SpA) is routinely performed with conventional radiography limited by superposition in the thoracic segments and radiation exposure. The objective was to assess the reliability of MRI compared to conventional radiography in depicting morphologic vertebral lesions in patients with axial SpA. Forty patients diagnosed with axial SpA were included in this cross-sectional study. Patients underwent MRI of the whole spine with T1-weighted and TIRM sequences in the sagittal plane and conventional radiography of the cervical and lumbar spine in lateral projections. Morphologic changes (syndesmophytes and erosions) in the anterior vertebral endplates on MRI and conventional radiography were independently evaluated by two radiologists. Inter-modality and interobserver agreement were calculated using Cohen’s Kappa.

**Results:**

Inter-modality agreement was low for cervical and lumbar syndesmophytes and erosions (κ ≤ 0.2 ± 0.07–0.1). Interobserver agreement on conventional radiography was highest for cervical and lumbar anterior syndesmophytes/bridging (*κ* = 0.92 ± 0.02–0.03). Syndesmophytes in thoracic anterior vertebral units were the most frequent MRI finding with a high interobserver agreement (*κ* = 0.83 ± 0.05).

**Conclusions:**

In imaging morphologic changes in the spine in patients with axial SpA, MRI was shown to be not an equivalent substitute but a complementary imaging modality to conventional radiography. Conventional radiography seems superior to depict morphologic cervical and lumbar vertebral changes compared to MRI, whereas MRI may visualise morphologic lesions in the thoracic spine.

## Key points

- MRI is more a supplementary modality than substitution to radiography in axial spondyloarthritis.
- Variation of findings of vertebral morphologic changes between MRI and radiography is high.
- MRI can detect thoracic morphologic spinal changes that might be missed with radiography.

## Background

Spondyloarthritis (SpA) belongs to a group of inflammatory rheumatic diseases often associated with human leukocyte antigen HLA-B27 and presenting with enthesitis, osteitis and arthritis.

Axial skeleton manifestations are predominant in axial SpA and less frequently observed in psoriatic arthritis, inflammatory bowel disease associated with SpA, reactive SpA and undifferentiated SpA [1–4]. Depending on cumulative disease activity, early inflammation and later new bone formation leads to progressive ankylosis of the sacroiliac joints and vertebral column.

Morphologic changes seen on conventional radiography differentiate between non-radiographic and radiographic SpA. Radiographic progression in the latter is commonly measured using the modified Stoke Ankylosing Spondylitis Spinal Score (mSASSS) [5–8]. To calculate mSASSS, SpA-associated structural lesions in the anterior endplates of the cervical and lumbar vertebrae are analysed in lateral conventional X-ray projections [6]. Diagnostic delay of up to 7 years in symptomatic patients suffering from lower back pain is mainly attributed to the delayed manifestation of structural lesions on conventional radiography [9, 10]. Mean radiographic progression of axial SpA is estimated to be around 30% in 10 years [11–13], with syndesmophytes being considered the best predictors of radiographic progression [14].

In recent decades, the value of magnetic resonance imaging (MRI) in diagnosing axial SpA and monitoring disease activity during therapy has increased. MRI has the potential to detect bone marrow oedema and osseous changes such as fatty lesions, erosions and sclerosis prior to their manifestation on conventional X-rays, while avoiding radiation exposure. Bone marrow oedema as a sign of osteitis in axial SpA and monitoring of therapeutic effects are best depicted with MRI [15–22]. In contrast to lateral conventional X-rays, MRI does not lead to structural superposition of the thoracic spine [18, 23]. However, conventional radiography seems to better depict structural damage, such as syndesmophytes, because of better spatial resolution than MRI [24]. General recommendations for the use of MRI in the diagnostic work-up and monitoring of disease activity in axial SpA therefore remain controversial [25, 26].

The purpose of the present study was to compare the capability of MRI compared to conventional radiography for depicting morphologic changes in the spine in patients with axial SpA.

## Materials and methods

### Study population

Forty consecutive patients (10 female, 30 male) of 18 years of age or older who had been diagnosed with axial SpA [4] and experienced symptoms for more than 5 years were included in this cross-sectional study conducted between November 2016 and April 2018.

We excluded patients with claustrophobia (as a contraindication to MRI examination), patients missing conventional radiography and patients who were unable to lie still for the duration of an MRI examination. Patients with another contraindication to MRI (e.g. non-MRI conditional metallic foreign bodies or implants), patients with a Cobb angle > 20° [27], pregnant women and patients refusing informed consent were also not included in the study.

The local institutional board approved all protocols before initiation of the trial. The study fulfilled Good Clinical Practice guidelines, and before their inclusion, all patients gave written informed consent.

### Clinical examination and functional analysis

Consultant physicians in the Department of Rheumatology and Immunology with 5–25 years of experience obtained the informed consent and patient characteristics and performed the clinical examinations. Each patient's functional deficits were assessed by the Bath Ankylosing Spondylitis Functional Index (BASFI) [28] and the Bath Ankylosing Spondylitis Metrology Index (BASMI) [29]. SpA disease activity was measured by the Bath Ankylosing Spondylitis Disease Activity Index (BASDAI) [30], based on the number of swollen and painful joints. A blood sample was taken to determine C-reactive protein (CRP) and blood sedimentation rate (BSR) to calculate the Ankylosing Spondylitis Disease Activity Score (ASDAS) as a composite score of disease activity.

### Imaging and image interpretations

Conventional plain X-ray images of the cervical and lumbar spine in lateral projections were obtained in every patient no more than 6 months prior the MRI examination. The modified Stoke Ankylosing Spondylitis Spine Score (mSASSS), as an established scoring method, was used to assess the spine, scoring the anterior superior and inferior vertebral corners from the second cervical to the first thoracic and the last thoracic to first sacral vertebra as follows: normal (0), erosion/sclerosis/squaring (1), syndesmophyte (2), bony bridging (3), with the total score ranging from 0 to 72 [6]. Anterior superior and inferior vertebral corners were merged to a spinal unit starting with the anterior inferior corner of the second cervical vertebra. Findings according to the mSASSS in these spinal units were recorded if one or both corners showed morphologic changes. For conventional radiography, 12 ventral vertebral units (6 cervical and 6 lumbar) per patient were analysed, amounting to a total of 480 units (240 cervical and 240 lumbar units) in all patients. The findings were classified and finally scored according to the mSASSS, as described above.

MRI was performed with a 3.0 Tesla scanner (Skyra, Siemens Healthineers). The spine was scanned with T1-weighted turbo spin echo (TR 600 ms, TE 8.5 ms, flip angle 160 degrees, slice thickness 3.5 mm, voxel size 1 × 1 × 3.5 mm) and Turbo-Inversion Recovery Magnitude (TIRM; TR 2800.0 ms, TE 35.0 ms, TI 220 ms, flip angle 137 degrees, slice thickness 3.5 mm, voxel size 1.2 × 1.2 × 3.5 mm) sequences in sagittal planes to assess morphologic changes. Anterior superior and inferior vertebral corners were merged to a spinal unit starting with the anterior inferior corner of the second cervical vertebra to the anterior superior corner of the first sacral vertebra, comprising the cervical, thoracic and lumbar spine. Recorded changes in the anterior vertebral corners in MRI were erosions and syndesmophytes.

We did not include sclerosis and squaring of the anterior vertebral endplates in the evaluation of the spinal MRI since no reliable data on these morphologic lesions are available with this method. For MRI, 23 ventral units of the vertebral column (6 cervical, 11 thoracic and 6 lumbar) per patient were analysed, amounting to a total of 920 analysed units (240 cervical, 240 lumbar and 440 thoracic units) in all patients.

All cervical and lumbar radiographic and MRI spine images were analysed independently in a random order by two radiologists (radiographic image interpretation: reader 1 and reader 2; MRI interpretations: reader 1 and reader 3) with 4 to 20 years of experience in musculoskeletal imaging. Image interpretation was performed on a workstation with a high-resolution monitor. The readers were blinded to patients’ demographic and clinical characteristics and the reports of the other imaging modalities. Results were anonymised and stored in two separate files on hard drives at the facility.

### Statistical analysis

Patient characteristics were tabulated as absolute numbers with means/medians and standard deviations/ranges as appropriate. Disease-specific scores and laboratory findings were recorded as descriptive statistics.

For inter-modality agreement between conventional radiography and MRI, first Cohen’s κ for findings in conventional radiography and MRI were obtained for reader 1 and reader 2 vs. reader 3 and then the average of the resulting κ values was calculated for each spinal segment. The comparisons were made for erosions and syndesmophytes in both modalities and for syndesmophytes/bridging in conventional radiography versus syndesmophytes or erosions in MRI in the cervical and lumbar spine, since the mSASSS does not consider the thoracic spine. As intervertebral spaces are clustered within patients, quantile-based confidence limits and statistical tests for Cohen’s κ were calculated by using a bootstrap sample size of 2000 from the patients [31].

To assess interobserver agreement between conventional radiography and MRI findings, Cohen’s κ was calculated for the spinal segments. For conventional radiography, interobserver agreement was calculated for erosions, syndesmophytes/bridging, sclerosis and squaring of anterior vertebral units in the cervical and lumbar spine. On MRI, interobserver agreement was calculated for erosions and syndesmophytes of anterior vertebral units in the cervical, thoracic and lumbar spine.

## Results

We included 40 patients with axial SpA (30 male and 10 female) and a disease duration of 15.1 ± 10.0 years (mean ± SD) in our study. The patients’ median age was 53.0 (range 32.0–69.0) years, and the mean duration of symptoms reported was 22.3 ± 9.7 years. More details on patient characteristics are presented in Table 1.

**Table 1:** Patient characteristics

Sex, female/male	10/30
Age	53.0 (32.0–69.0) years
HLA-B27 positive, *n* (%)	34 (85.0)
Duration of symptoms	22.3 (9.7) years
Time since diagnosis	15.1 (10.0) years
BASDAI	3.3 (1.7)
BASFI	2.6 (1.7)
BASMI	2.8 (1.6)
ASDAS	1.8 (0.8)
CRP	2.5 (0–20.9) mg/L

Duration of symptoms, time since diagnosis, BASDAI, BASFI, BASMI and ASDAS are presented with mean (standard deviation); age and CRP are presented with median (range)

### Comparison of radiographic and MRI findings

**Fig. 1:**
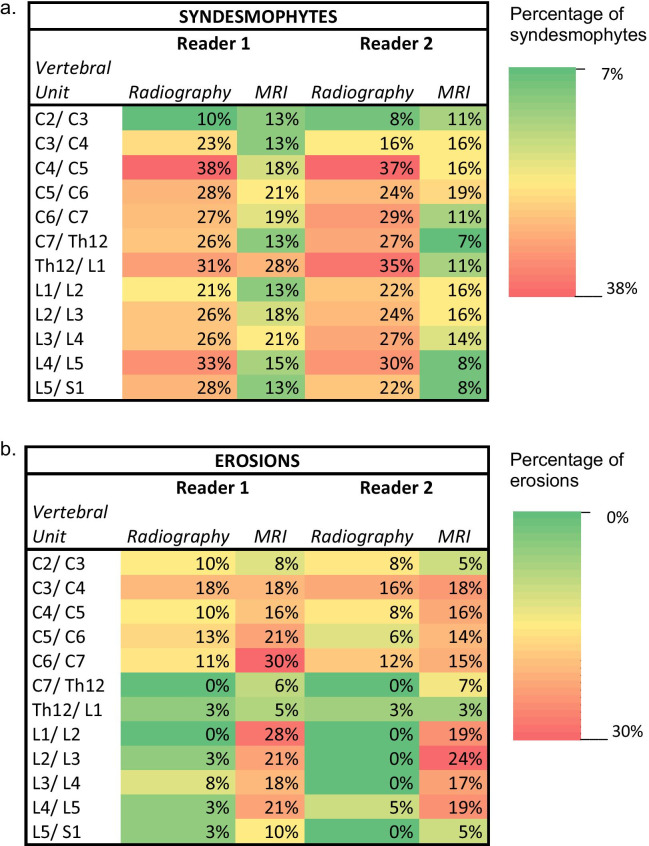
Heatmaps of reported syndesmophytes (**a**) and erosions (**b**) found on radiography and MRI for each reader. **a** Percentages in the heatmaps represent the proportion of reported findings in the study population. **b** Percentages in the heatmaps represent the proportion of reported findings in the study population

Inter-modality agreement between conventional radiographic imaging and MRI in general was very low (Table 2). Heatmaps presenting the frequencies of spinal erosions and syndesmophytes are shown in Fig. 1.

**Table 2:** Agreement between radiographic and MRI findings for erosions and syndesmophytes/bridging in the cervical and lumbar spine

*N* = 40	Cohen’s Kappa	
Radiography versus MRI	Cervical spine	Lumbar spine
Erosions versus erosions	0.13 ± 0.10	0.03 ± 0.05
Syndesmophytes versus syndesmophytes	0.13 ± 0.09	0.15 ± 0.10
Syndesmophytes and bridging versus syndesmophytes	0.20 ± 0.09	0.20 ± 0.07
Syndesmophytes and bridging versus erosions	0.20 ± 0.09	0.10 ± 0.08

The given Cohen’ Kappa is an average value of two readers per modality ± standard deviation

First, we evaluated and compared the distribution of erosions seen on conventional radiography and on MRI. We found very low levels of agreement between these two modalities at the cervical and lumbar vertebral units, with κ values of 0.13 ± 0.10 for the cervical spine and 0.03 ± 0.05 for the lumbar spine.

**Fig. 2:**
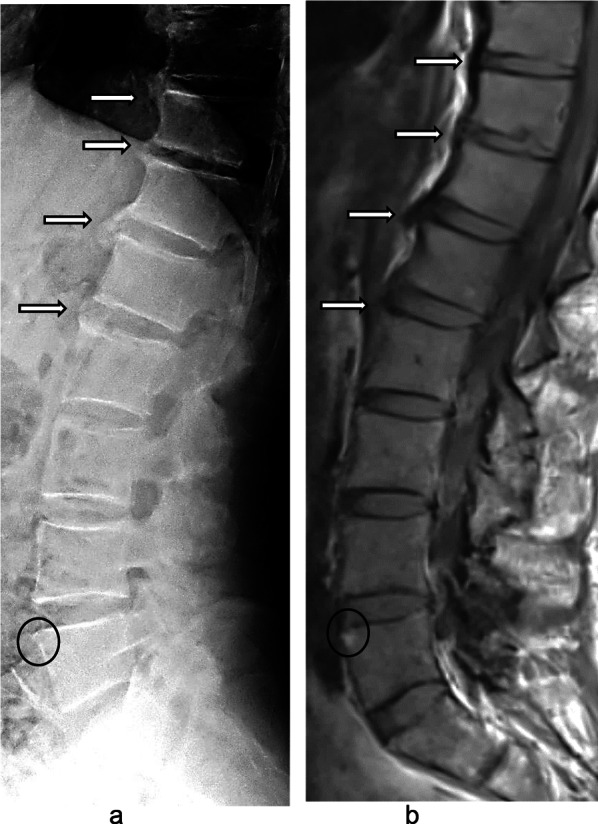
Radiography (**a**) and T1-weighted MRI (**b**) of the lower thoracic and lumbar spine. Images in the sagittal plane in the same patient (male, 53 years old) diagnosed with axial SpA. The extent of osseous chronic changes at the anterior vertebral corners is depicted in more detail with radiography than with MRI (white arrows), while a presumed disease-related fatty transformation at the ventral vertebral corner of L5 is only appreciated with MRI (black circles)

When analysing the distribution of syndesmophytes at the level of vertebral units radiographically and on MRI, we again found very low levels of agreement between the two modalities. The κ value was 0.13 ± 0.09 for cervical and 0.15 ± 0.01 for lumbar units. Figure 2 shows an example of lumbar syndesmophytes imaged with conventional radiography and MRI. When comparing the combination of both syndesmophytes and bridging on radiography to the presence of syndesmophytes on MRI, κ values were slightly higher, at 0.20 ± 0.09 for cervical units and 0.20 ± 0.07 for lumbar units. Since the same morphologic vertebral changes may have different appearances depending on the imaging modality, we compared the prevalence of reported syndesmophytes/bridging in conventional radiography with reported erosions in MRI. Cohen’s κ was 0.20 ± 0.09 for the cervical and 0.10 ± 0.08 for the lumbar spine.

### Morphologic radiographic findings

With radiographic imaging, the presence of lumbar syndesmophytes was the most common finding (averaging 2.4 per patient), followed by the presence of cervical syndesmophytes (averaging 2.0 per patient). The interobserver agreement in detecting syndesmophytes was high, with κ values of 0.92 ± 0.03 for cervical syndesmophytes and 0.92 ± 0.02 for lumbar syndesmophytes.

Bridging of vertebral units was much more frequently present in the lumbar spine (averaging 1.4 per patient) than in the cervical spine (averaging 0.6 per patient), with high interobserver agreement and κ values of 0.90 ± 0.08 for cervical ankylosis and 0.92 ± 0.03 for lumbar ankylosis.

Erosions were predominantly detected in the cervical spine (averaging 0.6 per patients) in comparison with the lumbar spine (averaging 0.1 per patient), with good interobserver agreement for the cervical spine (*κ* = 0.79 ± 0.09) and the lumbar spine (*κ* = 0.54 ± 0.22).

Interobserver agreement levels were lower for squaring of the vertebral bodies (average frequency: 1.4 per patient in the cervical spine, 0.7 per patient in the lumbar spine) and sclerosis (average frequency: 0.5 per patient in the cervical spine, 0.9 per patient in the lumbar spine): κ values were 0.48 ± 0.07 for squaring of the cervical vertebral bodies, 0.48 ± 0.11 for squaring of the lumbar vertebral bodies, 0.39 ± 0.10 for sclerosis of the cervical spine and 0.44 ± 0.10 for sclerosis of the lumbar spine.

The correlation coefficient for the mSASSS between the two readers of conventional radiography was 0.86.

### Morphologic MRI findings

**Fig. 3:**
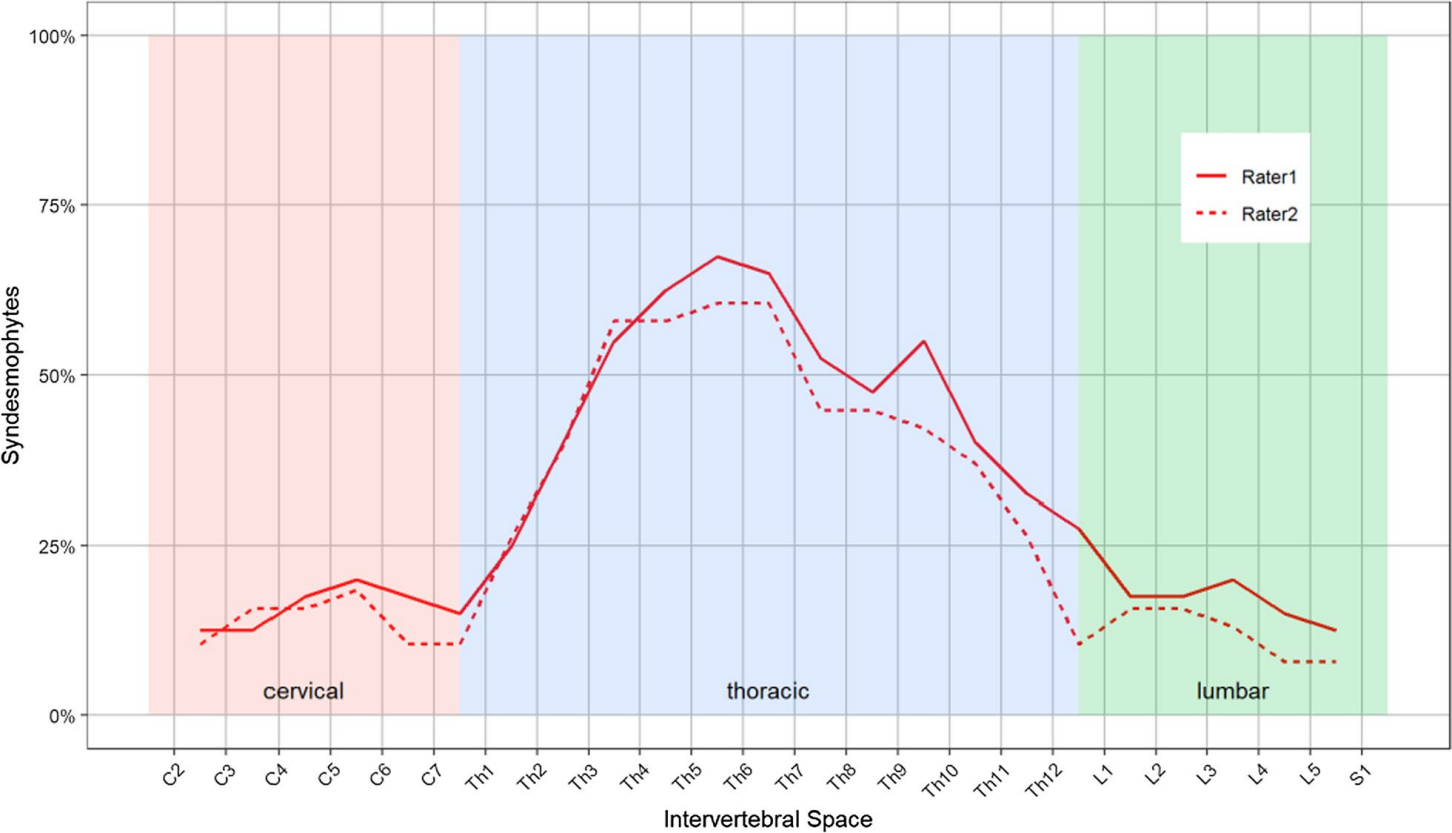
Frequencies of anterior vertebral syndesmophytes diagnosed with MRI. Syndesmophytes reported on MRI were identified most frequently in the thoracic spine, a vertebral portion that is not considered with mSASSS

**Fig. 4:**
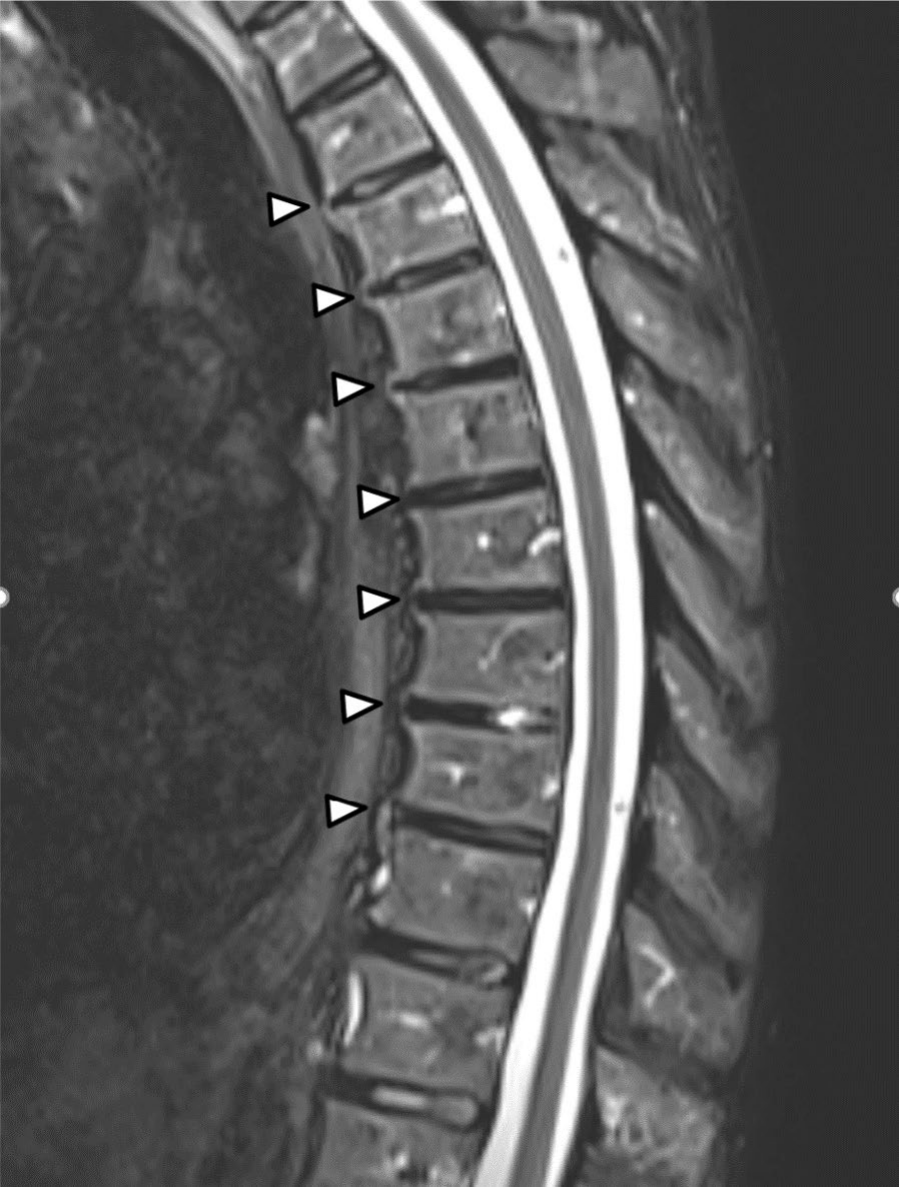
MRI (TIRM sequence) of the thoracic spine. Image in the sagittal plane in a patient (male, 61 years old) diagnosed with axial SpA. Multiple thoracic anterior syndesmophytes are depicted (white arrowheads)

At MRI, anterior syndesmophytes were the most frequently observed morphologic finding. A total of 254 spinal anterior syndesmophytes were identified on MRI among the 40 patients in the study. Notably, syndesmophytes were most frequently reported in the thoracic spine (Fig. 3), which is not included in the determination of mSASSS by conventional radiography because of superimposition in lateral projections. An exemplary MRI image of thoracic anterior syndesmophytes in a patient with SpA is presented in Fig. 4. Specifically, 196 syndesmophytes (an average of 4.9 per patient) were found in the thoracic spine, 30 (averaging 0.8 per patient) in the lumbar spine and 28 (averaging 0.7 per patient) in the cervical spine. κ values for interobserver agreement of the two MRI readers for anterior vertebral syndesmophytes were 0.83 ± 0.05 for the thoracic, 0.75 ± 0.11 for the cervical and 0.71 ± 0.08 for the lumbar spine.

Anterior erosions were also seen most frequently in the thoracic spine (averaging 1.4 per patient) and less frequently in the lumbar (averaging 0.9 per patient) and the cervical spine (averaging 0.8 per patient) with good interobserver agreement (κ values: for the cervical spine, 0.86 ± 0.07; for the thoracic spine, 0.69 ± 0.11; and for the lumbar spine, 0.76 ± 0.10).

## Discussion

In the evaluation of morphologic findings in patients with SpA, we found low inter-modality agreement between conventional radiography and MRI of the cervical and lumbar spine and high interobserver agreement on conventional radiography and MRI. The most common finding on MRI was the presence of syndesmophytes in the thoracic spine, a region that is not routinely assessed during the evaluation of radiographic progression of axial SpA.

Development of new syndesmophytes indicates ongoing inflammation and insufficient therapeutic intervention in patients with SpA and is a predictor of irreversible ankylosis of the spine with impaired spinal mobility [13]. In addition, spinal osteoproliferation is linked to decreased quality of life and work disability [32]. Therefore, imaging of structural vertebral lesions may guide therapeutic intervention and is part of the treat to target concept to improve outcome in axial SpA [33]. Currently, progression of bone formation as a hallmark of axial SpA is measured with the mSASSS evaluating the ventral portions of the vertebral body of the cervical and lumbar spine with conventional radiography [34].

Besides lack of radiation exposure, MRI has advantages over conventional radiography when it comes to depicting inflammatory changes such as bone marrow oedema that is impossible to detect at conventional radiography. On the other hand, due to better spatial resolution, conventional radiography may perform better than MRI in displaying subtle cortical bony changes in vertebrae [24]. Recent MRI developments with increased spatial resolution offer promising opportunities to close this gap in diagnostic capability. Krabbe et al. demonstrated the feasibility of visualising morphologic changes in the vertebral column using a 3-Tesla MRI-based scoring system for erosions and bone formation including ankylosis, but they did not compare their findings with the results of conventional radiography [35]. Only one prior study compared the value of conventional radiography and MRI for detecting morphologic changes in the spine in patients with axial SpA. In that study, three different scores to evaluate morphologic changes, including the mSASSS, were compared to the ASspiMRI-c as a MRI-based score for morphologic changes in the spine. The authors found a good correlation of the MRI score with the newly developed Berlin score and the Bath Ankylosing Spondylitis Radiographic Score (BASRI) but not with the mSASSS, indicating that the mSASSS may not reflect the whole spectrum of morphologic changes in axial SpA [36].

In our trial, syndesmophytes in the cervical and lumbar spine were more often found on conventional radiography than on MRI. This could be explained by conventional radiography having both better spatial resolution for detecting fine bony extension without MRI-detectable bone marrow and greater robustness against artefacts. As a result, inter-modality agreement was low when comparing findings of syndesmophytes/bony bridging on radiography to findings of syndesmophytes on MRI; indeed, our results suggest that inter-modality agreement between conventional radiography and MRI is lower than that found in a similar trial elsewhere [36].

Erosions, on the other hand, were more often observed in the cervical and lumbar spine on MRI than on conventional radiography. Since radiography has better spatial resolution than MRI, which makes it theoretically more appropriate for detecting vertebral erosions, this result was unexpected. However, the differences in sensitivity for detecting structural osseous lesions could lead to variations in interpretations between X-rays and MRI: What were interpreted as small syndesmophytes on radiography could have been described as structural changes on MRI that were more compatible with erosions than with syndesmophytes. This hypothesis is further supported by our inter-modality comparisons, where the κ value for erosions reported by radiography versus MRI was lower than that of syndesmophytes/bridging on radiography versus erosions on MRI.

The most frequent finding in MRI was anterior syndesmophytes in thoracic spine. Because of superimposition in lateral projections, the thoracic spine is not evaluated with conventional radiography when using the mSASSS. Syndesmophytes, however, are known strong predictors of radiographic progression in axial SpA [14], so MRI may offer a diagnostic imaging modality for assessing the thoracic spine to complete the evaluation of morphologic spinal changes in these patients.

Low-dose CT protocols could be a novel approach in the evaluation of structural osseous changes in the spine and sacroiliac joints in patients with SpA [37, 38]. In contrast to conventional radiography, low-dose CT of the spine is devoid of superposition in thoracic segments and can assess the dorsal vertebral corners and facet joints, but at the cost of higher patient’s radiation exposure and the likelihood of overscoring of syndesmophytes [39–41]. Another potential imaging modality for the evaluation of vertebral structural lesions is digital tomosynthesis, a low-dose X-ray imaging technique to generate tomographic images, that may depict structural lesions at the vertebral corners and facet joints more accurately than conventional radiography [42, 43]. Yet there are no recommendations for the implementation of low-dose CT or digital tomosynthesis in the assessment of vertebral structural lesions in SpA. Future trials may evaluate the performance of MRI compared to low-dose CT scans or digital tomosynthesis of the vertebral column in detecting structural changes in axial SpA. In addition, results of MRI-based synthetic CT for the detection of structural lesions in the sacroiliac joints seem promising and suggest further investigations of this modality in the evaluation of the spine in SpA [44].

The limitations of this study include the small number of patients. Still, it is the largest prospective trial in a blinded setting to date comparing the diagnostic strengths and interobserver variability of conventional radiography and MRI for detecting morphologic changes in the spine in axial SpA.

Image interpretation is inevitably subject to variation when it comes to reporting subtle morphologic bony changes like erosion or incipient syndesmophytes formation, and there is currently no established gold standard for the detection of spinal structural lesions in SpA. We therefore calculated the interobserver agreement, which was high for both conventional radiography and MRI, suggesting reliability of our image evaluations.

Moreover, our study does not include analysis of radiographic changes in the thoracic spine seen on conventional radiography, since we focused on the mSASSS as the most widely used method to evaluate structural changes in the spine.

In conclusion, the results of this study suggest that MRI is no equivalent alternative to conventional radiography, but conventional radiography and MRI are complementary imaging modalities to assess spinal disease-specific changes in axial SpA. In the cervical and lumbar spine, conventional radiography seems superior to MRI in depicting morphologic changes, whereas MRI may visualise highly prevalent morphologic changes in the thoracic spine in addition to inflammatory vertebral lesions.

## Data Availability

The data sets used and/or analysed during the current study are available from the corresponding author on reasonable request.

## Abbreviations

ASDAS: Ankylosing Spondylitis Disease Activity Score
BASDAI: Bath Ankylosing Spondylitis Disease Activity Index
BASFI: Bath Ankylosing Spondylitis Functional Index
BASMI: Bath Ankylosing Spondylitis Metrology Index
BASRI: Bath Ankylosing Spondylitis Radiographic Score
BSR: Blood sedimentation rate
CRP: C-reactive protein
HLA: Human leukocyte antigen
mSASSS: Modified Stoke Ankylosing Spondylitis Spinal Score
SpA: Spondyloarthritis
TIRM: Turbo inversion recovery magnitude
